# Educating for equity, navigating reality: community health nurses implementing interventions to eradicate Chhaupadi in Karnali, Nepal

**DOI:** 10.1186/s12912-025-04012-w

**Published:** 2025-11-04

**Authors:** Animesh Ghimire, Mamata Sharma Neupane

**Affiliations:** 1 Sustainable Prosperity Initiative Nepal, Bhimsengola, Thulo Kharibot, Baneshwor-31, Kathmandu, Nepal; 2 School of Nursing, Chitwan Medical College, Bharatpur-5, Kailashnagar, Chitwan, Nepal; 3 School of Public Health, Chitwan Medical College, Bharatpur-5, Kailashnagar, Chitwan Nepal

**Keywords:** Gender equity, Social change, Stakeholder participation, Capacity building, Health equity, Health education, Health policy, Human rights

## Abstract

**Background:**

Chhaupadi—a customary practice of menstrual seclusion rooted in notions of ritual impurity—persists in parts of Nepal, including Karnali Province, despite legal prohibition. Contemporary evidence has associated this practice with adverse health risks and psychosocial distress among women and girls. Community Health Registered Nurses (CHRNs) operate on the frontline of health services and are strategically positioned to deliver educational interventions addressing Chhaupadi. However, their specific experiences in designing and implementing such interventions, as well as the challenges and perceived effectiveness within this complex context, remain critically under‑explored.

**Methods:**

This qualitative study employed an interpretive description approach. In-depth, semi-structured interviews were conducted with eleven CHRNs, selected based on purposive sampling for their active involvement in Chhaupadi eradication efforts. Initial participant recruitment utilized convenience sampling through self-registration via advertised flyers, which was supplemented by snowball sampling to reach more participants. An overarching maximum variation strategy guided the final sample composition to ensure diverse perspectives. Data were analyzed inductively through thematic analysis.

**Results:**

Four interconnected themes emerged, depicting the operational realities faced by CHRNs: (1) Capacity Building at the Frontline, illustrating their pedagogical adaptations and culturally sensitive communication strategies; (2) Addressing Gaps and Navigating Complex Realities, highlighting the necessity for direct CHRN’s interventions and the intricacies of stakeholder engagement amid concealed practices; (3) Gauging Ripple Effects, revealing the profound challenges in measuring training effectiveness and defining success within an environment characterized by systemic ambiguities; and (4) Navigating System Constraints, detailing the impact of resource scarcity, policy–practice gaps, enduring cultural resistance, and workforce instability.

**Conclusion:**

This study reveals the multifaceted interplay between CHRN’s agency, community dynamics, and broader systemic factors in efforts to eradicate Chhaupadi. The results emphasize the need for targeted CHRN’s support, culturally congruent intervention design, enhanced resource allocation, and inter-sectoral collaboration to ensure effective policy enforcement and sustainable program delivery. By illuminating the frontline challenges of translating health education policy into practice, these findings contribute critical evidence for advancing health equity and gender equality in Nepal and similarly affected contexts, reinforcing the essential role of Community Health Registered Nurses as skilled facilitators and strategic navigators of social change.

**Clinical trial number:**

Not applicable.

## Introduction

Chhaupadi, a deeply entrenched socio-cultural tradition prevalent in western Nepal, represents a significant violation of human rights and a harmful form of gender-based violence [1]. Rooted in enduring beliefs regarding menstrual impurity, this practice mandates the seclusion of women and girls during menstruation and the postpartum period, often banishing them to rudimentary, unsafe shelters separate from their homes and communities [2]. This isolation is accompanied by numerous prohibitions—restricting entry to homes, kitchens, and temples, as well as contact with people, livestock, and food sources [3]. Women and girls subjected to Chhaupadi face heightened risks of physical ailments such as reproductive tract infections, hypothermia, and injuries from environmental hazards or animal attacks, alongside psychosocial distress that manifests as loneliness, fear, anxiety, shame, and depression [4]. Furthermore, the practice perpetuates gender inequality, disrupts education, and increases vulnerability to violence, undermining the overall health and well-being of affected individuals and communities [5]. The persistence of Chhaupadi is inextricably linked to patriarchal norms and specific interpretations within Hindu traditions that equate menstruation with impurity, reinforced by pervasive myths and superstitions that resist change [6].

The burden of Chhaupadi is particularly pronounced in Karnali Province, situated in Nepal’s remote northwestern region. Nepal’s status as a lower‑middle‑income country (GNI per capita USD 1,090) is not a stand‑alone statistic; it signals constrained public resources and institutional capacity that, when layered onto Karnali’s mountainous geography and sparse infrastructure as one of the country’s seven provinces, intensify service‑delivery gaps and weaken enforcement of rights‑based reforms [7, 8]. Karnali ranks as the nation’s least developed province, with a population exceeding 1.5 million [9], 51.2% experiencing multidimensional poverty, and 28.9% living below the national poverty line; its per capita income of USD 606 remains substantially lower than national averages. These structural barriers—pervasive poverty, limited access to education and services, gendered power relations, and infrastructural inadequacies—create conditions in which harmful traditional practices such as Chhaupadi persist and their consequences are magnified [10]. In turn, these constraints curtail the reach of formal enforcement and heighten the need for community‑embedded educational responses that connect policy to everyday practice.

In response to the documented harms of Chhaupadi, Nepal outlawed the practice via a Supreme Court directive in 2005 and criminalized it under the National Criminal Code in 2017 [11, 12]. Yet eradication remains elusive because implementation at the community level is uneven: routine enforcement is rare, monitoring is largely complaint‑driven and episodic, local authorities often lack clear operational protocols and dedicated resources, and fear of social sanction encourages concealment [10]. Consequently, the law functions as a normative anchor but not a reliable deterrent in many rural settings. It is within this enforcement gap that the frontline health workforce—particularly Community Health Registered Nurses (CHRNs) working alongside Female Community Health Volunteers (FCHVs)—steps in to translate statute into everyday change by delivering rights‑informed health education, brokering dialogue with families and community leaders, escalating high‑risk cases, and mentoring volunteers to sustain behavior change.

Prior work on FCHVs has established their unparalleled reach into households and their central role in health promotion; however, three persistent constraints recur in this literature: wide variation in formal education and training, limited positional authority when confronting sensitive socio‑cultural norms, and the inherent precarity of volunteer roles [13, 14]. These limitations help explain why degree‑qualified CHRNs are necessary partners in addressing Chhaupadi. CHRNs are Registered Nurses who complete a four‑year Bachelor of Science in Nursing (BScN) after Year 12 and are licensed by the Nepal Nursing Council (NNC) [15–18]. This professional status confers clinical credibility and public trust, enabling CHRNs to secure engagement from families, men and elders, traditional healers, and local officials, and to translate health and legal guidance into negotiated, context‑appropriate action. In practice, CHRNs’ roles have expanded within primary health care to include designing and adapting educational materials, mentoring and supervising FCHVs, and escalating high‑risk cases—functions that complement, rather than replace, the community embeddedness of FCHVs [19]. By foregrounding CHRNs’ distinctive contributions—professional authority, pedagogical adaptation, and systems navigation [20]—this study addresses a critical gap left by FCHV‑centred scholarship and clarifies why CHRN deployment matters for shifting entrenched socio‑cultural norms around menstruation.

Despite the strategic significance of CHRNs, especially in their role of training and guiding FCHVs, a significant gap remains in the literature concerning the synergistic relationship between CHRNs and FCHVs [21]. While existing research has documented the prevalence and consequences of Chhaupadi and extensively explored the role of FCHVs [22, 23], there is a lack of in-depth understanding regarding the specific experiences, perspectives, and challenges faced by CHRNs in designing, implementing, and evaluating educational interventions aimed at eradicating Chhaupadi within the complex settings of Karnali. Their unique position—bridging formal health system knowledge with community realities while managing training responsibilities—warrants focused investigation. This becomes more important as previous research has highlighted that rural women are hesitant to contact FCHVs due to their perceived incompetence [24], as most of these volunteers have minimal to no formal education. Therefore, this study aims to explore the experiences and perspectives of CHRNs in Karnali Province, Nepal, regarding the implementation and perceived effectiveness of educational interventions designed to eradicate the practice of Chhaupadi.

Situated within Nepal’s decentralized primary health system—where constrained budgets, volunteer‑dependent outreach, uneven enforcement of rights‑based laws, and gendered power hierarchies shape what is possible [25]—CHRNs occupy a distinctive meso‑level position. As degree‑qualified, NNC‑registered professionals embedded in local facilities, CHRNs translate policy into practice, broker accountability across households, FCHVs, and local authorities, and lend clinical legitimacy to sensitive norm‑change efforts. Against this political–economic backdrop, this study asks: How do CHRNs in Karnali Province design, implement, and assess educational interventions to counter Chhaupadi within conditions of resource scarcity, weak enforcement, and gendered power relations, and in what ways do their practices mediate the policy–practice gap?

## Methods

### Study design

This study employed interpretive description as its primary methodological approach within a constructivist framework, complemented by a qualitative descriptive orientation that influenced both data collection and presentation [26–28]. Interpretive description was chosen to generate practice-relevant insights into how CHRNs design and deliver educational responses to Chhaupadi in complex, context-specific settings. The complementary descriptive approach underscored a commitment to low-inference, plain-language accounts that closely reflect participants’ meanings, while the interpretive method provided the analytical basis to move beyond mere surface description toward more nuanced pattern-level explanations with direct relevance for public health nursing and policy.

### Setting and participants

This study was conducted within the community settings served by government health facilities, primarily Health Posts (HP) and Primary Health Care Centers (PHCCs), in the Jajarkot and West Rukum districts of Karnali Province, Nepal. Karnali Province, encompassing 10 districts (Dailekh, Dolpa, Humla, Jajarkot, Jumla, Kalikot, Mugu, Salyan, Surkhet, and Western Rukum), is characterized by its profound geographical remoteness, challenging mountainous terrain, and its status as Nepal’s least developed province. It registers significantly lower human development indicators and higher poverty rates compared to national averages [29, 30], factors which can exacerbate the persistence of harmful traditional practices. Jajarkot and West Rukum districts were strategically selected for this research due to the well-documented, continued high prevalence of Chhaupadi within their communities [3], thus providing a critical context for exploring CHRN-led intervention strategies and their inherent challenges.

Participants were CHRNs who were registered with the Nepal Nursing Council (NNC) and holding a minimum qualification of a four-year Bachelor of Science in Nursing (BScN). Inclusion criteria specified that participants must: (1) be currently employed as a CHRN within a government community health setting in either Jajarkot or West Rukum district for at least one year; (2) possess direct experience in educating FCHVs and/or community members regarding Chhaupadi; and (3) have been involved in planning or implementing health programs aimed at addressing Chhaupadi or related maternal and child health issues within their catchment area. A total of eleven community health registered nurses (CHRNs) (*N* = 11) meeting these criteria participated in the study.

Participant recruitment was initiated through purposive sampling [31] facilitated by flyers displayed in prominent locations accessible to CHRNs within Health Posts and PHCCs across the Jajarkot and West Rukum districts. These flyers detailed the study’s purpose, the voluntary nature of participation, assurances of confidentiality, the specific inclusion criteria, and provided a QR code for self-registration. CHRNs who identified themselves as meeting all eligibility criteria were invited to self-register their interest in participating. This initial self-registration process yielded seven CHRNs aligning with the convenience sampling strategy [32], and the ethos of voluntary participation [33]. The Principal Investigator (AG) subsequently contacted these individuals to verify their eligibility against all inclusion criteria, all of whom were confirmed as suitable and consented to participate.

To achieve the target sample size and potentially include CHRNs who may not have accessed or responded to the flyers, a supplementary snowball sampling technique was then employed [34]. Following their interviews, a subset of the initial seven participants were asked to suggest other CHRNs within the eligible districts who also met al.l inclusion criteria and whom they believed could offer valuable, potentially diverse, insights regarding Chhaupadi interventions. This referral process facilitated the recruitment of the remaining four participants to ensure maximum variation within the participants [35].

Efforts were made to ensure the final sample of eleven CHRNs encompassed individuals with differing years of experience working in Karnali, varied roles where applicable, and those working in geographically distinct areas within the two selected districts. This strategy aimed to enhance the breadth and depth of the data collected, ensuring a range of experiences and perspectives on the complexities of addressing Chhaupadi. All participation was voluntary, and informed consent was obtained from every participant.

### Data collection

Data were collected between October 2024 and February 2025 through face-to-face, in-depth, semi-structured interviews conducted by the primary investigator (AG). To foster openness, no prior professional relationship existed between the investigator and the participants. Interviews were conducted at private locations convenient for the participants, such as within their health facility, to ensure privacy and confidentiality. Written informed consent was obtained from each participant prior to commencing the interview, following a thorough explanation of the study’s objectives, the voluntary nature of participation, confidentiality assurances, and the right to withdraw at any time without consequence.

Interviews were audio-recorded with permission and conducted in Nepali and English. The language of the interview was determined by participant preference at the outset, with participants free to speak entirely in Nepali, entirely in English, or to code-switch between the two as needed. The interviewer mirrored participants’ language use and explicitly encouraged code-switching for clinical, legal, or culturally specific terms to maximize precision and comfort. Language choice and any within-interview switching were documented in field notes to contextualize meaning and inform subsequent translation and analysis. It is important to note that English is the primary language for curricula, textbooks, reference materials, and reading resources mandated for instruction and study across all educational levels in Nepal, from nursery to tertiary education [36]. The semi-structured interview guide (Table 1) was developed based on a literature review from previous studies done on FCHVs [4, 5, 22, 23] and refined following expert consultation with PG, a Nepalese health policy expert. It explored CHRNs’ experiences with designing and adapting educational materials, training FCHVs, direct community engagement strategies, perceived effectiveness of interventions, encountered challenges, stakeholder interactions, monitoring practices, and navigating systemic factors related to Chhaupadi. Probing questions were used to elicit rich, detailed narratives. Participants were encouraged to ask questions throughout the process. Interviews lasted approximately 45 to 60 min each.

Both authors are registered nurses and academics familiar with the Nepalese healthcare context and fluent in Nepali and English. The first author (AG) transcribed all audio recordings verbatim. Nepali portions were translated into English by AG, and the accuracy of transcription and translation was cross-checked by the second author (MSN) on a random subset of transcripts to ensure accuracy. Where interviews included code-switching, transcripts preserved the original Nepali and English segments verbatim; key emic terms (e.g., *goth*, *dhami/jhankri*) were retained with brief English glosses to maintain cultural nuance. Transcripts were de-identified using codes (CHRN 1-CHRN11). As part of the member checking process, participants were later provided with summaries or relevant transcript sections to validate the accuracy of the recorded information.

**Table 1:** Semi-structured interview guide outline

Interview domain	Illustrative questions
Background & Role	- Can you describe your current role as a CHN and your main responsibilities related to community health education? - How long have you worked in this district/Karnali?
Experience with Chhaupadi Interventions	- What kind of educational activities or interventions regarding Chhaupadi have you been involved in? - Can you describe your experience training FCHVs on this topic?
Training Strategies & Adaptation (for FCHVs/Comm.)	- How do you adapt official guidelines or materials for FCHVs or community members? - What methods do you find most effective for training/education on sensitive topics like Chhaupadi?
Direct Community Engagement & Challenges	- Are there situations where you intervene directly in the community regarding Chhaupadi? Why? - What challenges do you face when discussing Chhaupadi with community members or FCHVs?
Stakeholder Interaction	- How do you interact with local leaders, traditional healers, or other stakeholders regarding Chhaupadi?
Perceived Effectiveness & Monitoring	- How effective do you feel these educational efforts are? How do you gauge success or progress? - What are the challenges in monitoring the impact of these interventions?
Systemic Factors & Support	- What resources (or lack thereof) affect your ability to address Chhaupadi? - How does existing policy or lack of enforcement impact your work? - What support do you receive/need?
Concluding Thoughts	- Is there anything else you feel is important regarding your experience working on Chhaupadi eradication?

### Data analysis

An inductive thematic analysis approach was employed to analyze the transcribed interview data [37], guided by the principles outlined by Braun and Clarke [38]. Consistent with interpretive description, analysis proceeded concurrently with data collection in iterative cycles of coding, constant comparison, analytic memoing, and progressive focusing to generate practice-relevant explanations. First, both researchers independently read and re-read the transcripts to achieve familiarization with the depth and breadth of the content. During familiarization, reflexive notes were kept to document early interpretive hunches about clinical, organizational, and socio-cultural processes shaping CHRNs’ work. Second, initial codes were systematically generated from the data, capturing semantic and latent meanings related to the CHRNs’ experiences and perceptions regarding Chhaupadi interventions. AG conducted the initial coding, meeting regularly with MSN to discuss, refine, and compare codes, ensuring consistency. In keeping with interpretive description and reflexive TA, these meetings functioned as critical interpretive dialogues aimed at deepening, challenging, and extending meaning rather than calculating inter‑coder agreement. Third, codes were collated, reviewed, and grouped into emerging themes and sub-themes based on patterns of meaning. We actively sought variation and disconfirming (negative) cases and compared within and across settings to strengthen explanatory accounts. Fourth, these potential themes were reviewed and refined against both the coded data extracts and the entire dataset to ensure they accurately captured the participants’ narratives. Analytic memos and data displays (e.g., thematic maps and matrices) were used to examine relationships among codes and themes and to test emergent propositions against the full dataset. Fifth, clear definitions and labels were established for each final theme and sub-theme. Theme development prioritized interpretive, practice-oriented explanation (how and why CHRNs’ practices operated in context) over topic summaries, aligning with interpretive description’s goal of generating actionable insights for care and policy. Rather than pursuing “data saturation,” this study focused on achieving thematic saturation within the dataset [39], reflecting on Braun and Clarke’s [40] view of reflexive thematic analysis as an interpretive, iterative process. Consistent with interpretive description, analytic adequacy was judged by interpretive sufficiency—i.e., when the evolving account cohered, was grounded across cases (including negative cases), and ceased to yield substantively new practice‑relevant insights [41]. In this approach, meaning is understood as being constructed through engagement with the data rather than simply “discovered” within it. Consequently, decisions about the number of participants or when to cease data collection are inherently situated, context-dependent, and cannot be predetermined solely by the notion of saturation. Indeed, additional participants may well have offered new or divergent perspectives, and the research design acknowledged that richness and variability in participants’ lived experiences are essential for a nuanced understanding of the phenomenon under study. Iterative returns to the dataset throughout analysis ensured that interpretive moves remained grounded in participants’ accounts while moving beyond description to pattern-level explanation, which was carried out manually without the use of any software. Table 2 illustrates an example of the data analysis process, tracing the progression from raw meaning units to final themes.

**Table 2:** Example of data analysis process

Meaning Unit (Illustrative Excerpt)	Code	Category	Merged Sub-theme	Theme
**Theme 1 Examples**				
1a. “The district module uses words the FCHVs don’t understand… I have to use simple pictures I draw myself… discuss it in our local dialect, relate it to stories they know.”	Adapting materials; Simplifying language; Local dialect use; Storytelling	Training material adaptation; Culturally sensitive communication methods	1.1 Pedagogical adaptation and cultivating sensitive communication	Theme 1: Capacity Building at the Frontline
1b. “We developed a set of picture cards showing a girl going to school… another helping cook… asking them, ‘What problems do you see if she stays in the goth?’ It sparked so much discussion.”	Visual aids; Participatory learning; Prompting discussion; Relatable scenarios	Tool development for engagement; Interactive training techniques	1.2 Contextual training Tools for engagement and motivation	Theme 1: Capacity Building at the Frontline
**Theme 2 Examples**				
2a. “I had to go myself because the FCHV was scared of the father… my uniform and position sometimes carry a weight that the volunteer doesn’t have.”	Direct intervention; CHN authority; FCHV limitations; Addressing intimidation	Reasons for direct CHN involvement; Overcoming volunteer barriers	2.1 Direct CHRN interventions: Addressing Implementation Gaps	Theme 2: Addressing Gaps and Navigating Complex Realities
2b. “They say ‘no Chhaupadi here’, but then later, you hear whispers… They hide it. They fear being reported, being shamed, or even boycotted by neighbours.”	Hidden practice; Fear of reporting; Social stigma; Community concealment	Challenges in assessment; Dynamics of concealment; Navigating social pressures	2.2 Navigating Stakeholder Dynamics and Hidden Practices	Theme 2: Addressing Gaps and Navigating Complex Realities
**Theme 3 Examples**				
3a. “We do the training…they nod. But how do I really know if they understood, if they are using the right messages? We don’t have a system for observing them regularly.”	Uncertainty of impact; Lack of follow-up; No observation system	Difficulty verifying skill transfer; Monitoring FCHV practice deficiencies	3.1 Monitoring and Tracking Training Effectiveness/ Deficiencies	Theme 3: Gauging Ripple Effects
3b. “Sometimes, the best sign is informal. An FCHV tells me fewer women are complaining of infections… Or a schoolteacher mentions more girls are attending classes. These small stories… feel more real.”	Anecdotal evidence; Indirect health indicators; School attendance as proxy	Using alternative progress markers; Subjective success indicators	3.2 Informal Indicators and Success Amidst Systemic Ambiguity	Theme 3: Gauging Ripple Effects
**Theme 4 Examples**				
4a. “To do proper training for all FCHVs, or to visit remote villages myself… it needs budget. For travel, for materials… Often, the Chhaupadi budget is tiny or non-existent.”	Resource constraints; Lack of funds; Budget limitations for activities	Impact of financial scarcity; Barriers to program implementation	4.1 Resource Scarcity and Policy and Practice Divide	Theme 4: Navigating System Constraints
4b. “This belief… it’s deeper than just habit. It’s tied to religion, to fear of misfortune… Changing something so deeply woven into the culture… that is the biggest challenge.”	Deep-rooted beliefs; Religious influence; Fear of misfortune; Cultural identity	Nature of cultural opposition; Challenges to normative change	4.2 Cultural Resistance and Workforce Challenges	Theme 4: Navigating System Constraints

### Rigor and trustworthiness

To ensure the trustworthiness of the findings, this study adhered to established criteria for qualitative rigor: credibility, dependability, confirmability, and transferability [42].

Credibility was enhanced through several strategies. Member checking was integral [43]; preliminary thematic summaries, including key findings and supporting anonymized quotes, were shared with all participants via their preferred contact method. Participants were invited to review these summaries to verify if the interpretations resonated with their experiences and accurately reflected their intended meanings. They were encouraged to provide feedback or suggest modifications. All eleven participants responded, providing affirmations and minor clarifications that were incorporated into the final analysis, thereby strengthening the correspondence between participant accounts and the researcher’s interpretations. Prolonged engagement was facilitated by the interviewer’s immersion in the data collection phase over several months [44], allowing for the development of rapport and a deeper understanding of the context.

Dependability was addressed through a transparent and meticulously documented research process. The semi-structured interview guide was developed following a comprehensive literature review (searching PubMed, CINAHL, Google Scholar using keywords: “Chhaupadi,” “menstrual seclusion,” “Nepal,” “community health nurse,” “health education,” “intervention,” “Karnali,” limited to English language publications in the last 15 years) and refined based on consultation with an external expert in Nepalese health policy. An audit trail, comprising interview transcripts, audio recordings, field notes, coding frameworks, analysis memos, and records of team discussions, was maintained throughout the study, allowing for methodological scrutiny.

Confirmability was promoted through rigorous reflexivity [45]. The research team, comprising nursing academics with insider knowledge of the Nepalese healthcare system, engaged in ongoing reflexive journaling and dialogue. Regular team meetings provided a critical space to discuss emerging interpretations, challenge assumptions grounded in personal or professional experiences, and ensure that themes were demonstrably grounded in the participants’ narratives. For instance, initial interpretations of CHRN’s frustration might have been biased towards workload; however, reflexive discussion guided by transcript evidence helped clarify the distinct roles of systemic barriers versus personal capacity. The detailed audit trail further supports confirmability by making the analytical steps transparent.

Transferability was enhanced by providing a detailed description of the study context (Karnali Province, specific districts, and community setting), the participants’ characteristics (qualifications, experience, and roles), and the data collection and analysis processes. While the findings are specific to the experiences of these CHRNs in these particular settings, the detailed contextual information allows readers to assess the potential resonance and relevance of the themes to other remote, resource-limited settings in Nepal or globally where similar health interventions targeting entrenched socio-cultural practices are implemented.

### Ethical considerations

Ethical approval for this study was obtained from the Nepal Health Research Council (NHRC) [Approval Number: 332/2024] and District Health Offices in Jajarkot and West Rukum districts prior to commencing data collection. All participants were provided with detailed information sheets in Nepali and English explaining the study’s purpose, procedures, potential risks and benefits, confidentiality measures, and data usage. Written informed consent was obtained from every participant before the interview. Participation was entirely voluntary, and participants were explicitly informed of their right to withdraw at any stage without penalty or impact on their employment.

## Results

### Participant characteristics

The study included eleven Community Health Registered Nurses (CHRNs; coded CHN1–CHN11) working in community settings across Jajarkot (*n* = 6) and West Rukum (*n* = 5) districts of Karnali Province (Table 3). The cohort was exclusively female, reflecting the gender distribution of Nepal’s nursing workforce [46]. All participants held a Bachelor of Science in Nursing (BSc Nursing); ages ranged from 25 to 38 years (median 27). Two were concurrently pursuing a Master of Public Health degree. Tenure in Karnali ranged from 1 to 6 years (median 3), with most participants having fewer than five years’ service, consistent with known retention challenges in remote postings [47]. To characterize diversity relevant to work and social roles, four participants reported urban origins and seven rural origins, and five were married while six were single at the time of the interview. These characteristics provide contextual nuance for interpreting participants’ accounts of designing and delivering Chhaupadi‑related educational interventions within resource‑constrained communities.

**Table 3:** Participant characteristics table

Participant	Gender	Age (Years)	Marital status	Highest qualification	Years working in Karnali	Geography (District of practice)	Setting
CHN1	Female	26	Married	BSc Nursing	3	Jajarkot	Community
CHN2	Female	27	Single	BSc Nursing	5	West Rukum	Community
CHN3	Female	25	Single	BSc Nursing	2	Jajarkot	Community
CHN4	Female	38	Married	BSc Nursing	6	West Rukum	Community
CHN5	Female	31	Single	BSc Nursing	4	Jajarkot	Community
CHN6	Female	35	Married	BSc Nursing	5	West Rukum	Community
CHN7	Female	25	Single	BSc Nursing	1	Jajarkot	Community
CHN8	Female	30	Married	BSc Nursing	4	Jajarkot	Community
CHN9	Female	26	Single	BSc Nursing	3	West Rukum	Community
CHN10	Female	27	Married	BSc Nursing	2	Jajarkot	Community
CHN11	Female	25	Single	BSc Nursing	1	West Rukum	Community

### Findings

**Fig. 1:**
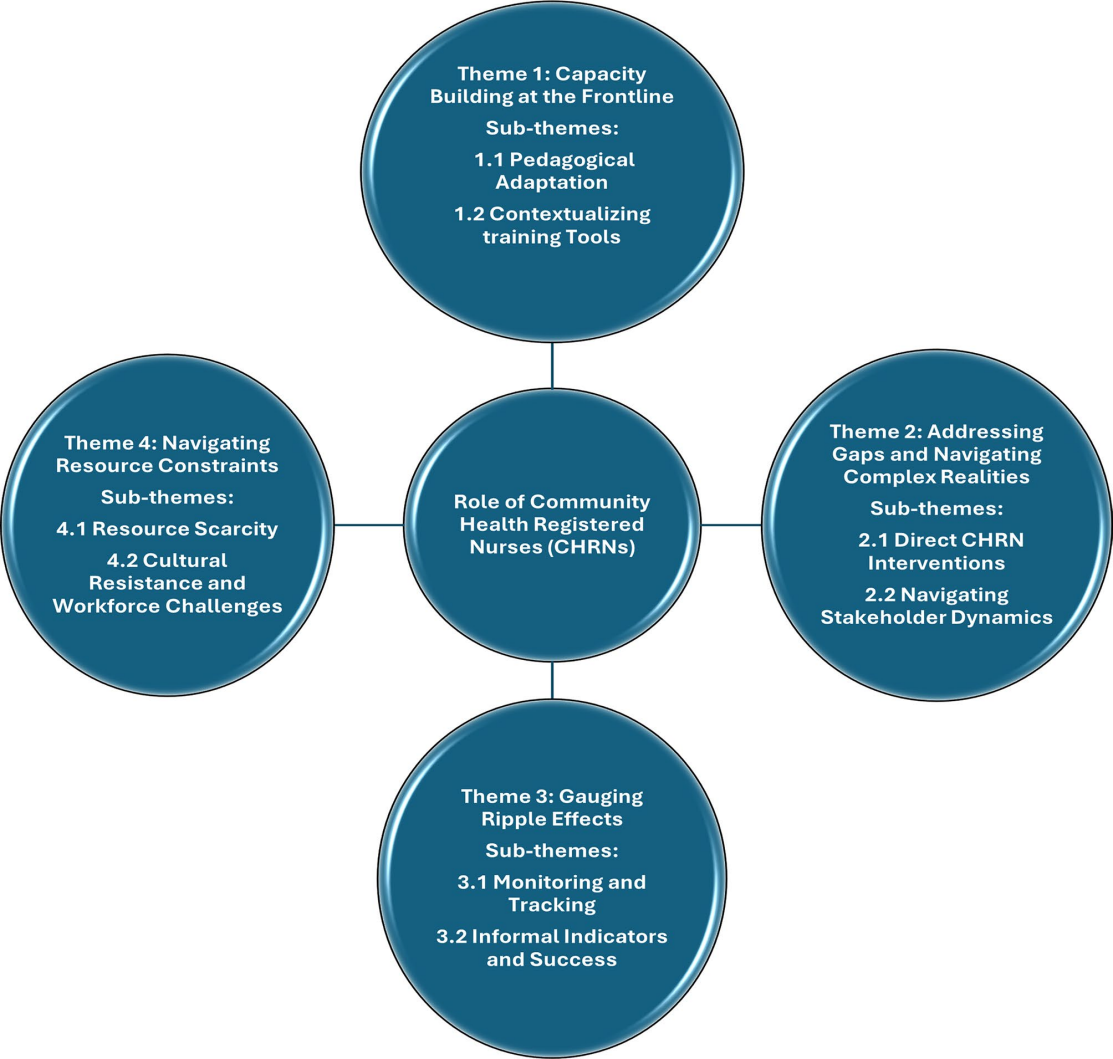
Thematic map illustrating the central role of Community Health Registered Nurses (CHRNs) in mobilizing Female Community Health Volunteers (FCHVs) to eradicate Chhaupadi

The analysis of eleven semi-structured interviews with Community Health Nurses (CHRNs) in Karnali Province revealed a multifaceted landscape of experiences related to educational interventions targeting the eradication of Chhaupadi. These findings underscore the CHRNs’ central role not only as direct educators within communities but also as trainers and supervisors of Female Community Health Volunteers (FCHVs). The data highlight the CHRNs’ adaptive and innovative frontline strategies in confronting entrenched cultural practices, resource scarcity, and systemic gaps in policy implementation and monitoring. Figure. 1 outlines the four overarching themes and their corresponding sub-themes.

## Theme 1: Capacity building at the frontline

### Pedagogical adaptation and cultivating sensitive communication

Several CHRNs emphasized the necessity of translating technical health information into accessible, culturally resonant materials for FCHVs who often have limited formal education. One participant noted:

*We get the educational modules from the district*,* but most of the time the language is too technical for the FCHV didis [older sisters/volunteers]. During our monthly meetings*,* I break it down in detail. We use simple drawings that I create myself*,* showing the inside of the body and explaining why bleeding occurs*,* why it’s a natural process*,* and not impure […] It feels less daunting*,* and by tailoring the content to their needs*,* they genuinely become more interested and motivated to tackle Chhaupadi back in their villages. This approach works because most of our didi’s [FCHVs] lack formal qualifications.* (CHRN 11)

Another CHRN highlighted role-playing exercises to develop empathetic and effective communication skills among FCHVs:

*Just drilling the FCHVs that ‘Chhaupadi is bad*,* Chhaupadi is illegal’ achieves little. They live in that community*,* amongst those families! So*,* we practice scenarios – intense role-playing. I’ll be a very resistant mother-in-law*,* invoking tradition*,* or a husband terrified of the gods’ wrath if they don’t send menstruating women to the chhau huts [menstrual huts]. The key learning? They must practice listening first*,* before reacting with facts[…] How do you ask sensitive questions gently? How do you discuss hygiene without inducing shame? It’s about arming them with the confidence to have these incredibly difficult conversations*,* ensuring they don’t alienate the very families they need to influence.* (CHRN 8)

Such experiential learning fosters critical “soft skills,” enabling volunteers to respond sensitively to deeply entrenched beliefs. By modeling empathetic listening and culturally attuned messaging, CHRNs help FCHVs navigate the fine line between advocating change and preserving their credibility within the community.

### Contextualizing training tools for engagement and motivation

Beyond oral instruction, participants described designing simple yet culturally resonant visuals:

*We made this set of picture cards […] simple drawings showing a girl attending school during her period*,* another helping her mother cook*,* another sleeping safely inside a warm room. During a meeting with FCHVs*,* we laid these out and asked*,* ‘Looking at these*,* what problems arise if she stays in the cold goth [hut] instead?’ It sparked a lively discussion*,* far more engaging than just my lecture. Those images resonated instantly; they saw their own lives reflected.* (CHRN 1)

By representing local realities and shifting focus from abstract principles to vivid, relatable scenarios, CHRNs reported higher engagement and deeper reflection among volunteers. These participatory methods also fostered a sense of ownership of the message, encouraging volunteers to incorporate newly gained insights into their subsequent community outreach.

Such training often leads to observable shifts in volunteer competence and confidence:

*I particularly remember one FCHV didi [older sisters/volunteers] very reserved at first […] After lots of one-on-one encouragement*,* she blossomed […] She finally convinced a particularly stubborn family to let their daughter sleep safely inside*,* in a separate room*,* not out in the shed.”* (CHRN10)

These “small wins” highlight how incremental yet meaningful progress can stem from sustained support and mentorship. CHRNs consistently described these transformative outcomes as a key motivator that validates their efforts and underscores the potential ripple effects of effective capacity-building.

## Theme 2: Addressing gaps and navigating complex realities

### Direct CHRN interventions: addressing implementation gaps

*The FCHV came to me*,* distressed. A family was forcing their daughter*,* who had a high fever*,* into a goth [menstrual huts]. The FCHV felt intimidated by the girl’s father and uncles. In such acute situations*,* just relying on the volunteer’s training isn’t enough. I had to intervene directly. Go there myself*,* speak firmly with the family head*,* detail the immediate medical risks*,* remind them of the law […] Sometimes*,* my professional status*,* even just the uniform*,* carries an authority […] which the volunteer cannot wield in that moment.* (CHRN 5)

When systemic barriers or community resistance outweigh the influence of volunteers, CHRNs must pivot from a supervisory to an active enforcement role. By using legal, medical, and professional authority, they can neutralize power imbalances and promptly address immediate health threats. In these encounters, nurses explicitly referenced Nepal’s legal framework outlawing Chhaupadi—the 2005 Supreme Court directive and its criminalization under the National Criminal Code (2017) [12]—to legitimise clinical advice and secure compliance. Most CHRNs described intervening under two recurring conditions: acute clinical risk (e.g., fever, hypothermia) and pronounced power asymmetries that limited FCHV influence.

Several participants also underscored the importance of tailoring messages to entire communities rather than exclusively targeting women:

*When I visit a village specifically for Chhaupadi awareness*,* I don’t just talk to women; I meet with the entire community—men*,* elders*,* religious leaders included. I come prepared with data from our hospital register showing women treated for reproductive tract infections linked to poor menstrual hygiene*,* and I discuss local cases of snake bites in goths*,* hypothermia*,* and sexual assaults. I frame it clearly: this is not ‘tradition versus modern ways’; this is a serious health and safety crisis impacting everyone. (CHRN 3)*

Such efforts position Chhaupadi as a broad public health concern, rather than a niche’ women’s issue, thereby helping to mitigate stigma and facilitating broader community engagement. Several participants framed this reframing as essential for engaging male decision‑makers and elders, reporting that inclusive meetings reduced stigma and opened space for dialogue across households.

### Navigating stakeholder dynamics and hidden practices

*It’s truly a constant*,* delicate dance. With the dhami/jhankri [traditional healer]*,* you start with respect*,* acknowledge their community role*,* and listen patiently. Then*,* you gently introduce how certain Chhaupadi restrictions might actually worsen the very illnesses they aim to cure*,* suggesting a collaborative approach. And then with the elected Ward Chairman [government officer]*,* the language shifts – it becomes about local development indicators*,* girls’ education targets*,* fulfilling legal obligations under the constitution. After this*,* I might need to consult the district hospital doctor about a severe uterine prolapse case clearly exacerbated by Chhaupadi practices*,* bringing that clinical weight back into the community dialogue. Each requires a different approach.* (CHRN 2)

CHRNs therefore employ flexible communication strategies, appealing to different authorities, highlighting legal frameworks, or drawing on local public health data, depending on the interlocutor. Across interviews, nurses reported tailoring appeals to Nepal’s constitutional commitments to equality and dignity, and to the statutory prohibition of Chhaupadi, while simultaneously negotiating with traditional authorities to avoid backlash. However, some families conceal noncompliance for fear of legal repercussions or social sanctions:

*You do a household visit and ask about Chhaupadi. The response is often immediate: ‘Oh no*,* didi*,* we abandoned that long ago.’ But weeks later*,* whispers surface*,* or an FCHV quietly tells you the adolescent girl was seen confined near the cowshed during her last menstruation. They hide it meticulously—afraid of being reported or shamed*,* or facing social boycott if others still practice it. This makes tracking genuine progress almost impossible; building deep*,* trusting relationships is far more crucial than ticking checklists.* (CHRN 6)

This phenomenon underscores the limitations of self-reported data in contexts where stigma is deeply entrenched into the community fabric. It reinforces the need for sustained rapport-building and trust, as well as the importance of alternative methods for monitoring genuine behavioral change. Most participants reported this pattern of outward compliance with covert continuation, complicating reliance on self-reporting. Accordingly, CHRNs prioritized trust-building, triangulated accounts through FCHVs and, where feasible, school or clinic records, and shifted monitoring emphasis from checklist counts to dialogue-based assessments of change.

## Theme 3: Gauging ripple effects

### Monitoring and tracking training effectiveness/ deficiencies

A recurrent concern among CHRNs was the insufficient follow-up to ensure FCHVs internalize and accurately disseminate key messages:

*We conduct the training sessions for the FCHVs; they seem engaged*,* they nod*,* they might take some notes. But honestly? How do I truly gauge whether they have grasped the core message*,* understood the nuances*,* and are accurately and confidently conveying it back in their own villages? There is no structured system for follow-up observations or receiving systematic feedback on their practice.* (CHRN 7)

Under current systems, evaluations often rely on numerical outputs (e.g., “number of Chhaupadi awareness sessions”) rather than comprehensive quality assessments. This disjuncture between formal metrics and real-world implementation impedes timely adaptations to training methodologies:

*Look at our monthly report format. It asks: ‘Number of Chhaupadi awareness sessions conducted.’ Fine*,* we count the sessions. But does that number tell you anything about the quality of the session? The depth of the discussion? The resistance encountered? The fact that half the village might still be practicing secretly? No. It’s just a tally mark. It paints a potentially misleading picture*,* failing to capture the real*,* complex story unfolding on the ground or whether attitudes are genuinely shifting beneath the veneer of compliance.* (CHRN 11)

Such process-oriented indicators risk obscuring the complexity of sociocultural transformations, providing a potentially misleading picture of actual progress.

### Informal indicators and success amidst systemic ambiguity

In this evaluative void, CHRNs turn to informal feedback and anecdotal evidence:

*Often*,* the most telling signs of progress aren’t in any official report. It’s when an FCHV casually mentions that fewer women in her cluster are complaining about reproductive infections after their periods. Or when a local schoolteacher remarks that girls’ attendance seems more consistent throughout the entire month now. These small stories*,* these ‘whispers of change’ filtering back through informal channels […] they often feel more authentic*,* more indicative of real shifts than the statistics we report upwards [higher authorities]. However*,* capturing and formally validating them is the challenge.* (CHRN 10)

While these subtle shifts offer valuable insights into evolving attitudes, they lack systematic documentation, making it difficult to measure impact or replicate effective strategies at scale. Participants also questioned top-down definitions of “success,” suggesting that immediate goals—such as dismantling the goth or seeing open dialogue about menstruation—are important stepping stones even if total eradication of the practice remains distant:

*Honestly*,* what constitutes ‘success’ in this fight? Is it simply that the FCHV delivered the required awareness message? Is it when one family finally dismantles their goth? Or is it achieving the district’s target of zero goths across the entire ward – something that feels miles away? Those top-down targets often feel utterly disconnected from the grinding*,* day-to-day reality we face. For me*,* sometimes success feels smaller*,* more personal: seeing a woman finally speak about menstruation without deep shame*,* or witnessing a husband begin to openly question the practice his father upheld. It’s slow*,* painstaking*,* step-by-step progress.* (CHRN 9)

Such reflections underscore the need for a more nuanced and context-specific approach to measuring progress, valuing incremental cultural shifts that may not be captured in standardized targets.

## Theme 4: Navigating resource constraints

### Resource scarcity and policy and practice divide

Nearly all participants cited chronic resource constraints—limited transportation budgets, shortage of teaching materials, and minimal incentives for volunteers—as a major barrier:

*To conduct meaningful*,* regular training for all the FCHVs across these hills*,* or for me to travel to that remote village where serious problems are reported*,* it all requires a budget. Fuel is expensive—sometimes you need to hire a jeep. You need clear visual aids*,* not worn‑out flipcharts. Even basic tea and biscuits during long sessions help keep volunteers engaged. But the specific budget line for Chhaupadi work is minuscule. We have the determination but lack the basic means. We have the determination but often lack the basic means.* (CHRN 6)

Furthermore, although Chhaupadi is criminalized, its enforcement on the ground remains sporadic:

*Yes*,* the law clearly states Chhaupadi is illegal. We are trained to tell people that*,* and we do. But the reality here? Who actually enforces it consistently? The police might get involved if there’s a death or a major incident reported in the media*,* but routine enforcement? Very rare. The local government representatives might make speeches condemning it*,* but tangible action or penalties are slow to materialize. So*,* the law often feels like a distant concept*,* not an immediate deterrent. It adds some weight to our argument*,* yes*,* but it doesn’t automatically compel change when people see little consequence for non-compliance.* (CHRN 4)

Participants explicitly situated this gap against Nepal’s legal framework—noting that, in practice, the law serves as a normative anchor rather than a reliable deterrent in rural settings. Accordingly, most nurses reported relying on persuasion, rights‑informed health education, and relationship-centred negotiation to secure day‑to‑day compliance, with legal references used strategically to legitimize clinical guidance rather than as an enforceable threat.

### Cultural resistance and workforce challenges

Participants uniformly recognized that Chhaupadi is interwoven with spiritual beliefs and generational norms, complicating efforts to change behavior:

*You have to understand*,* this belief [Chhaupadi] it runs deeper than mere habit or ignorance. It’s intricately tied to religious interpretations*,* deeply ingrained fears of divine punishment or misfortune falling upon the family*,* and generations following in the footsteps of their ancestors. You find even highly educated individuals*,* sometimes even other health workers*,* quietly adhering to or believing in the necessity of some form of separation. Changing a simple health behavior like handwashing is one thing; challenging something so fundamentally woven into cultural identity*,* into religion […] that’s the monumental task. It demands extraordinary patience and determination […].* (CHRN 8)

Most CHRNs emphasized that adherence cut across education levels and professional status, reinforcing the need for long‑term, culturally attuned strategies that avoid backlash while steadily shifting norms. The role of FCHVs is equally crucial yet precarious:

*Remember*,* the FCHVs are volunteers. They have fields to plow*,* cattle to tend*,* families to feed. Asking them to confront neighbors and relatives about something as sensitive as Chhaupadi is a tremendous burden*,* especially with almost no tangible support or compensation. If they lose motivation*,* become discouraged by resistance*,* or drop out because life is too hard*,* the community health network weakens and more of the burden falls back to us [CHRNs]. Their stability is key.* (CHRN 10)

This account highlights the precarious nature of volunteer‑based models, particularly for entrenched practices. FCHVs, who already shoulder domestic and economic responsibilities, risk burnout or disengagement if insufficiently supported; their withdrawal disrupts outreach and shifts pressure to the limited cadre of paid nurses. Across these themes, CHRNs described using ongoing mentorship and small tokens of recognition to sustain morale, while noting that such efforts remain vulnerable to funding volatility. Taken together, resource scarcity, sporadic legal enforcement despite formal prohibition, and volunteer precarity create a structural context in which CHRNs must continually balance persuasion, pedagogy, and limited policy leverage to maintain incremental progress.

## Discussion

The findings of this study underscore the multifaceted role that community health registered nurses (CHRNs) occupy as both implementers and catalysts for change in contexts where formal health directives intersect with deeply ingrained cultural norms. CHRNs’ adaptation of curricula, use of local dialects, creation of visual aids, and fostering of informal learning environments align with established principles of effective health pedagogy and communication in cross-cultural, low-literacy settings [48, 49]. This study’s finding contrasts with previous studies that have focused primarily on FCHV activities [1, 4, 23, 50, 51], failing to highlight the often-invisible intellectual and creative labor that CHRNs invest in equipping these frontline volunteers. The reported emphasis on experiential, dialogue-based training—particularly the implementation of role-play—moves beyond mere knowledge transfer towards fostering the communication skills necessary to negotiate resistance and stigma. This aligns with social norm theory, which contends that effective behavioral change hinges on altering collective perceptions, trust-building, and respectful engagement especially in societies where entrenched cultural beliefs and stigmatization prevails [52, 53].

One of the key findings form this study is the flexibility CHRNs demonstrate when direct intervention becomes necessary. Contrary to conventional cascade models that position supervisors as passive overseers [54, 55], the CHRNs in this study actively harness their clinical authority and local credibility to address acute risks and entrenched power imbalances. Their engagement of diverse community segments, including men, elders, government officials, and traditional healers, highlights a sophisticated public health approach that frames Chhaupadi as a community-wide concern rather than a narrowly defined “women’s issue.” Through these nuanced “stakeholder dances,” CHRNs leverage multiple forms of authority—legal, clinical, and socio-cultural—to foster collective accountability for women’s safety and well-being [56]. The accounts of concealed practices underscore how social desirability biases can distort accurate self-reporting, a phenomenon also observed in research on other harmful traditional practices, such as female genital mutilation [57, 58]. In the context of this study, these findings highlight the difficulties CHRNs face in accurately assessing and addressing Chhaupadi, as stigma, fear, or community pressure lead individuals to conceal or underreport its continued practice. Such concealment emphasizes the need for more nuanced, trust-based data collection and intervention strategies that account for the cultural sensitivity surrounding Chhaupadi.

Another critical dimension relates to the CHRNs’ unease about the fidelity and real-world impact of their FCHV trainings, owing to minimal follow-up mechanisms and an overreliance on superficial reporting metrics such as “number of awareness sessions”. This critique aligns with broader discussions in the public health literature that advocate for mixed-methods evaluations capable of capturing both quantitative outcomes and the nuanced, qualitative shifts necessary for dismantling harmful socio-cultural practices [59, 60]. The discrepancy between top-down targets, for example, absolute eradication of Chhaupadi and the more incremental progress that frontline workers value—such as dismantling a single goth [huts] or observing reduced stigma during community discussions—reveals a disconnect in how success is defined. Recognizing smaller, contextually significant achievements may be pivotal for sustaining motivation and refining strategies in the long-term change initiatives.

Finally, the persistent lack of resources, weak law enforcement, and the precarious reliance on unpaid FCHVs underscore the systemic vulnerabilities that hinder these interventions. The policy–practice gap surrounding the Chhaupadi prohibition law, in particular, echoes the challenges commonly reported when rights-based legislation confronts resistant local norms [10]. At its core, Chhaupadi remains entwined with fundamental belief systems related to purity and identity, indicating that conventional “knowledge deficit” models of change are insufficient [50, 51]. The prominence of volunteer fatigue and competing livelihood, demands targeted efforts to maintain momentum against a practice perceived by some as indispensable to cultural cohesion. Taken together, these findings highlight the necessity of multifaceted, resource-backed strategies that integrate culturally competent education, legal support, and community-driven monitoring [61, 62]. By centering interventions around the nuanced experiences and expertise of Community Health Registered Nurses, programs can more effectively address deeply entrenched cultural norms. Such an approach highlights the importance of robust supervisory systems, sufficient resources, and sustained professional support for the frontline workforce. Collectively, these investments form a strategic framework for driving meaningful and lasting change in communities where harmful practices persist.

### Implications for practice

The experiences of CHRNs in Karnali Province point to several practical steps for improving Chhaupadi eradication programs in low-resource, culturally complex settings. First, CHRNs themselves require enhanced training that goes beyond clinical knowledge of menstrual health to include culturally sensitive communication, community negotiation, stakeholder engagement, participatory teaching methods, and supportive supervision strategies for FCHVs. Establishing ongoing peer support and mentorship networks for CHRNs can strengthen their capacity to address Chhaupadi’s socio-cultural dimensions. Second, it is vital to co-develop context-specific educational materials in collaboration with both CHRNs and FCHVs, ensuring that interventions incorporate local linguistic and cultural nuances. Adequate resources must be allocated for producing and distributing these materials. Third, a structured, supportive supervision framework should enable CHRNs to provide regular feedback and hands-on mentorship to FCHVs, shifting the emphasis from administrative oversight to genuine capacity building. Fourth, programs should be inclusive of men, elders, religious figures, and traditional healers, framing Chhaupadi as a collective health and societal concern rather than an isolated women’s issue, with gender-responsive engagement of men and boys as gatekeepers and allies to interrupt the intergenerational transmission of patriarchal norms. Finally, monitoring must extend beyond basic quantitative indicators to include qualitative and indirect measures (e.g., school attendance, infection rates, observable changes in household practices) to capture more subtle shifts in attitudes and behaviors, especially when practices are concealed.

### Implications for policy

#### Immediate and short‑term policy actions

Strengthen policy enforcement and accountability: Provide regular, structured training for local authorities and law enforcement on the legal and socio‑cultural dimensions of Chhaupadi, coupled with clear protocols for reporting, follow‑up, and accountability. Employ trauma‑informed, gender‑responsive approaches that minimize punitive backlash and avoid driving practices underground.

Increase resource allocation: Earmark budget lines for training, supportive supervision, community outreach, transport, and educational materials for CHRNs and FCHVs. Introduce modest, transparent support mechanisms (e.g., stipends, travel allowances, recognition schemes) to sustain frontline motivation and engagement.

Promote intersectoral collaboration: Establish district‑level coordination mechanisms linking health, education, local government, law enforcement, and women’s development offices to align work plans, pool resources, and unify messaging. Explicitly engage men and boys across sectors (schools, youth groups, local governance, police) to challenge and reshape gender norms.

#### Longer‑term and structural policy reforms

Invest in enabling infrastructure: Integrate improvements in water, sanitation, and hygiene (WASH) for households and schools; strengthen supply chains for affordable menstrual products; and develop practical guidelines for safe, dignified at‑home alternatives to seclusion [63]. Embed these investments in local development plans and budgets.

Integrate Chhaupadi objectives into broader policy frameworks: Embed eradication goals within gender equality, adolescent health, education, and poverty‑reduction policies. Adopt gender‑responsive budgeting and monitoring with sex‑ and age‑disaggregated indicators and explicit metrics of norm change. Formalize CHRN roles in national staffing, training, and supervision standards, including curricula on social norms and culturally sensitive communication, and ensure predictable financing for outreach and supervision.

### Limitations

While this qualitative study provides in-depth insights into the perspectives of CHRNs in Karnali Province, several limitations warrant consideration. First, although the sample size (*N* = 11) allowed for detailed exploration and was deemed adequate for thematic saturation in this exploratory inquiry, it may not capture the full range of perspectives among all CHRNs. Second, the sensitive nature of Chhaupadi potentially introduced social desirability bias, whereby participants could have withheld or minimized views conflicting with the official anti-Chhaupadi stance, despite the researchers’ efforts to ensure rapport and confidentiality. Third, the researchers’ positionality—including their disciplinary background, gender, and insider/outsider status—necessarily influenced data collection, analysis, and interpretation. Reflexive practices were employed to mitigate these effects; however, they cannot be wholly eliminated. These limitations underscore the need for cautious interpretation of the findings and for further research involving larger or more diverse samples to deepen understanding of CHRNs’ roles and experiences in eradicating harmful cultural practices.

### Future research

The insights generated by this study open avenues for further investigation. Conduct longitudinal research to track changes in CHRN strategies, FCHV capacity, community practices, and health/social outcomes over time following specific interventions or policy changes. Complement qualitative findings with larger-scale quantitative surveys to assess the prevalence of Chhaupadi practices, including variations and concealment, measure attitudes among different community segments, and statistically evaluate the reach and perceived impact of interventions across wider areas. Design and rigorously evaluate specific educational or multi-component interventions, potentially comparing different models of CHRN training, FCHV support, or community engagement strategies. Conduct focused research exploring the perspectives, roles, and potential influence of men and boys in perpetuating or challenging Chhaupadi, and practical strategies for engaging them in eradication efforts. Investigate how Chhaupadi intersects with other dimensions of marginalization, such as caste, ethnicity, disability, and poverty, to understand differential impacts and tailor interventions accordingly.

## Conclusion

This study underscores the vital yet challenging role of Community Health Registered Nurses in combating Chhaupadi in Nepal’s Karnali Province. Positioned at the intersection of formal health systems and strongly held traditions, CHRNs demonstrate adaptability and commitment in translating policy goals into culturally aligned action. Their efforts reveal that eradicating Chhaupadi transcends the mere enforcement of legal bans; it requires sustained, multifaceted engagement that provides robust training and resources for the health workforce, fosters inclusive community dialogue, addresses root causes of poverty and marginalization, and ensures consistent institutional support. By reinforcing CHRNs’ capacity to act as facilitators, educators, and strategic navigators, stakeholders can strengthen the collective push to safeguard the health, dignity, and rights of women and girls. Achieving a Chhaupadi-free Nepal thus necessitates an enduring, coordinated, and culturally astute effort across all sectors, maintaining an active commitment to ongoing dialogue, research, and iterative policy refinement.

## Data Availability

The data supporting this study’s findings are available on request from the corresponding author. However, the data is not publicly available due to privacy or ethical restrictions.
